# Zn-URJC-12 Material Constituted of Two Different Organic Ligands for CO_2_ Valorization into Cyclic Carbonates

**DOI:** 10.3390/nano15131018

**Published:** 2025-07-01

**Authors:** Jesús Tapiador, Pedro Leo, Pablo Salcedo-Abraira, Antonio Rodríguez-Diéguez, Gisela Orcajo

**Affiliations:** 1 Chemical and Environmental Engineering Group, Escuela Superior de Ciencias Experimentales y Tecnología (ESCET), Universidad Rey Juan Carlos, c/Tulipán s/n, 28933 Móstoles, Spain; jesus.tapiador@urjc.es (J.T.); gisela.orcajo@urjc.es (G.O.); 2 Department of Inorganic Chemistry, University of Granada, Avda. Fuentenueva s/n, 18071 Granada, Spain; psalcedo@ugr.es (P.S.-A.); antonio5@ugr.es (A.R.-D.)

**Keywords:** MOF, cyclic carbonates, carbon dioxide, CO_2_ valorization, CO_2_ cycloaddition, Zn-based MOF

## Abstract

A novel metal–organic framework based on zinc ions, designated as Zn-URJC-12, has been synthesized and applied for the first time in the cycloaddition reaction between carbon dioxide and epoxides. This MOF is constructed from two different organic linkers: 5-aminoisophthalic acid and 4,4′-biphenyldicarboxylic acid. The framework features –NH_2_ functional groups coordinated to Zn(II) centers, as confirmed by single-crystal X-ray diffraction analysis. Zn-URJC-12 demonstrates exceptional chemical stability in polar organic solvents, such as methanol, while maintaining thermal stability up to 250 °C. The material exhibits high catalytic efficiency in the cycloaddition of CO_2_ with epoxides, achieving yields of 100% and 76% for propylene oxide and allyl glycidyl ether, respectively. Additionally, Zn-URJC-12 maintains its structural integrity and catalytic performance during five successive reaction cycles. These findings underscore Zn-URJC-12 as a promising heterogeneous catalyst for the valorization of CO_2_ into cyclic carbonates.

## 1. Introduction

Metal–organic frameworks (MOFs) are coordination compounds composed of one or more metal ions and organic ligands [1]. These materials are distinguished by their well-defined crystalline structures, high porosity, and remarkable versatility [2], as their properties can be meticulously adjusted by varying the metal centers or organic linkers within the framework [3]. In recent years, there has been a significant increase in the synthesis of novel MOFs [4,5,6], driven by their potential in diverse applications, such as magnetism [7], luminescence [8], drug delivery [9], and catalysis [10], among others. In particular, MOFs are emerging as promising heterogeneous catalysts for CO_2_ valorization, owing to their advantages over conventional heterogeneous and homogeneous catalysts [11].

Cyclic carbonates are organic compounds with a wide range of applications. These include their use as solvents in battery electrolytes and as intermediates in the pharmaceutical, biomedical, and plastics industries [12]. The most common synthetic route for producing cyclic carbonates typically involves high pressures and temperatures in the presence of homogeneous catalysts [13]. An alternative, more economical and sustainable approach is the cycloaddition reaction between CO_2_ and epoxides. However, for this reaction to proceed efficiently, the presence of both basic and Lewis acidic sites in the reaction medium is essential [14], and various heterogeneous catalysts have been explored for this purpose. Unfortunately, many of these systems have shown limited performance, often requiring harsh conditions—such as elevated pressures and temperatures—and, in many cases, the use of solvents to achieve acceptable yields [15,16,17]. MOF-based catalysts offer a promising solution, as their structures can incorporate both acidic and basic functional groups, enabling the one-pot synthesis of cyclic carbonates from CO_2_ and epoxides under mild conditions [18,19,20,21].

Various metal ions have been employed as Lewis acid sites within MOF structures [22]. In previous studies, Zn(II) and Co(II) have demonstrated superior catalytic activity in the cycloaddition of CO_2_ and epoxides, attributed to their higher Lewis acidity [21,23]. However, the metal center is not the sole determinant of catalytic performance; the organic linker also plays a crucial role, as it can introduce Lewis basic sites into the framework [24]. The basicity of the functional groups present on the linker is a key parameter for achieving high catalytic efficiency [19].

Valverde-González et al. [25] synthesized two novel Co-based MOFs incorporating tetrazole-functionalized linkers. These materials, Co-NDTz and Co-NDPhTz, achieved epichlorohydrin conversions of 63% and 73%, respectively, under mild conditions (50 °C, 3 bar CO_2_, 6 h), highlighting the beneficial effect of nitrogen-containing linkers on catalytic performance. Similarly, Shang et al. [26] reported two MOFs based on Dy(III) and Tb(III), constructed with carboxylate-based linkers containing oxygen atoms capable of acting as Lewis bases. These materials exhibited excellent catalytic activity in the cycloaddition of CO_2_ with propylene oxide, attributed to the synergistic interaction between the metal centers and the organic linkers. Dy-MOF and Tb-MOF achieved yields of 92.6% and 90.3%, respectively—significantly higher than those obtained using the corresponding metal salts or ligands alone. Further evidence of the importance of acid–base synergy was provided by Guo et al. [27], who developed a novel 2D Cu-based MOF featuring unsaturated copper sites and sulfonate (–SO_3_) groups. This combination of Lewis acidic and basic functionalities enabled the catalyst to achieve high yields in the cycloaddition of CO_2_ with various epoxides: 100% for propylene oxide, 98% for epichlorohydrin, 84% for 1,2-epoxy-3-allyloxypropane, and 66% for tert-butyl glycidyl ether, under reaction conditions of 80 °C and 25 bar for 5 h.

In many studies involving novel MOF materials, the frameworks are typically constructed using a single organic linker. However, mixed-ligand MOFs offer several advantages over their single-linker counterparts [28]. These include the ability to fine-tune pore size and surface area, incorporate diverse functional groups into the framework, and enhance structural flexibility [29]. Such flexibility may arise from variations in metal coordination environments or from the intrinsic properties of the organic linkers. As a result, mixed-ligand MOFs often exhibit improved characteristics, such as enhanced gas adsorption capacity and superior catalytic performance.

Mixed-ligand strategies can be classified based on the nature of the linkers involved. This may include the use of a single type of linker bearing different functional groups, or the combination of structurally distinct organic ligands [30]. For example, Kleist et al. [31] synthesized a modified version of MOF-5 by partially substituting 1,4-benzenedicarboxylic acid with 2-aminoterephthalic acid. The resulting mixed-linker MOF (MIXMOF) was evaluated as a heterogeneous catalyst in the cycloaddition of CO_2_ with propylene oxide, achieving a yield of 63%, compared to 44% for the parent MOF-5. This improvement was attributed to the presence of Lewis basic groups in the MIXMOF structure, which enhanced catalytic activity. Another example is provided by Manna et al. [32], who developed a series of Zr-based MOFs incorporating two different organic ligands with similar backbone structures: bipyridyl- (mBPV-MOF) and phenanthryl-functionalized (mPT-MOF) linkers, both coordinated with iridium. The resulting catalysts, mBPV-MOF-Ir and mPT-MOF-Ir, exhibited superior catalytic activity compared to the non-functionalized BPV-MOF-Ir. This enhancement was attributed to the presence of larger open channels in the mixed-linker MOFs, which facilitated substrate diffusion and improved overall catalytic performance.

The use of pyridyl-based ligands to enhance the structural stability of MOF materials has been well documented in the literature [33]. For instance, Le et al. [34] synthesized the MOF Cu_2_ (BDC)_2_ (BPY) and evaluated its performance as a heterogeneous catalyst in the oxidative coupling of terminal alkynes with N–H amides. The incorporation of the 4,4′-bipyridine ligand significantly improved the structural robustness of the framework, resulting in enhanced catalytic activity compared to conventional Cu-based catalysts. Another example was reported by Masoomi et al. [35], who synthesized two novel Co-based MOFs. The first, TMU-10, was constructed using a single organic linker, 4,4′-oxybisbenzoic acid (oba), while the second, TMU-12, incorporated both oba and pyrazine (PY) as linkers. These materials were tested as heterogeneous catalysts in oxidative desulfurization reactions. TMU-12 exhibited superior catalytic performance relative to TMU-10, an improvement attributed to the presence of the PY ligand, which introduced additional void space and altered the coordination environment around the Co^2+^ centers, thereby enhancing the material’s catalytic efficiency.

Although MOFs exhibit promising catalytic activity in the cycloaddition of CO_2_ to epoxides, several intrinsic limitations restrict their scalability for industrial applications [36]. A major challenge is the chemical and thermodynamic inertness of CO_2_, which requires highly active catalytic sites to activate it. Additionally, the structural integrity of MOFs may be compromised under reaction conditions, especially at high pressures and temperatures, which could result in partial framework degradation or loss of crystallinity [37]. Pore accessibility is another significant limitation because bulky epoxides may encounter steric hindrance within the confined cavities of the MOF, thereby reducing catalytic efficiency [36]. Additionally, catalyst reusability is a concern because reaction products can become trapped within the porous network, necessitating extensive washing or regeneration procedures. These additional steps are not economically feasible on an industrial scale [37]. Therefore, the rational design of MOFs with enhanced structural robustness, tailored pore architectures, and multifunctional active sites is essential to advance their practical implementation in CO_2_ valorization processes.

For this reason, in the present work, we propose the synthesis and characterization of a novel Zn(II)-based MOF, designated as Zn-URJC-12, constructed from two distinct organic ligands: 5-aminoisophthalic acid and 4,4′-biphenyldicarboxylic acid. This new MOF will be evaluated as a heterogeneous catalyst in the cycloaddition reaction between CO_2_ and various epoxides. Additionally, its catalytic stability will be assessed over five consecutive reaction cycles using epichlorohydrin as the model substrate.

## 2. Materials and Methods

All starting materials and solvents were purchased from Cymit Química S.L., Barcelona, Spain and used without further purification.

Synthesis of Zn-URJC-12: First, 0.1 mmol (0.029 g) of zinc nitrate hexahydrate, 0.1 mmol (0.018 g) of 5-aminoisophthallic acid, and 0.1 mmol (0.024 g) of 4,4′-byphenyldicarboxilic acid were mixed with 5 mL of DMF and 1 mL of H_2_O in a 20 mL vial and sonicated for 15 min. Then, this yellow solution was heated at 90 °C for 72 h and cooled to room temperature. Pink-colored crystals were separated by filtration, washed three times with DMF, and dried in air. Yield: 73% based on linker 5-aminoisopthallic acid. FT-IR: 3260 (m), 3194 (m), 3107 (m), 3046 (m), 1655 (sh), 1610 (sh), 1567 (s), 1471 (w), 1438 (w), 1352 (s), 1256 (w), 1155 (m), 1112 (m), 1003 (w), 958 (m), 898 (w), 855 (w), 808 (w), 768 (m), 737 (m), 704 (w), and 675 (w) cm^−1^.

Single-Crystal X-Ray Diffraction. Single-crystal X-ray diffraction data were collected on a Bruker D8 VENTURE diffractometer (Bruker, Ettlingen, Germany) equipped with Mo Kα radiation (λ = 0.71073 Å) and a Photon III detector. The temperature during data collection was maintained at 100 K using a dry nitrogen cryostream (Oxford Cryostream 700, Oxford Cryosystems Ltd., Oxford, UK). Data reduction was performed using the APEX3 software suite, and absorption corrections were applied accordingly. The crystal structure was solved and refined using the SHELX software package [38,39] within the Olex2 (v1.5) interface [40]. All non-hydrogen atoms were refined anisotropically, while hydrogen atoms were placed in calculated positions and refined using idealized geometries. Crystallographic data (excluding structure factors) have been deposited with the Cambridge Crystallographic Data Centre under CCDC reference number 2449688. These data can be obtained free of charge from the CCDC (12 Union Road, Cambridge CB2 1EZ, UK; Fax: +44-1223-335033; Email: deposit@ccdc.cam.ac.uk).

Physicochemical Characterization Techniques. ^1^H-NMR spectra were collected with a Varian Mercury Plus spectrometer (Agilent, Santa Clara, CA, USA) at 400 MHz using trimethyl silane as an internal standard. FID files were processed using MestRe-C software version 4.9.9.6. The chemical shifts (δ) for 1H spectra, stated in ppm, are referenced to the residual proton signal of the deuterated chloroform. The chemical Shifts for ^13^C spectra are referenced to the signal from the carbon of the deuterated solvent. Powder X-ray diffraction (PXRD) patterns were obtained in a Philips XPERT PRO (Eindhoven, The Netherlands) using CuΚα (λ = 1.542 Å) radiation with a 0.01 step and 10 s of accumulation time between steps. In all the cases, the samples were grounded to avoid the effects of preferred crystal orientation. Fourier transform-infrared spectra (FT-IR) were recorded for powder samples in a Varian 3100 Excalibur Series spectrometer (Agilent, Santa Clara, CA, USA) with a resolution of 4 cm^−1^. Simultaneous thermogravimetry and derivative thermogravimetry analyses (TGAs) were carried out under an air atmosphere with a Mettler-toledo DSC-TGA Star System device (Mettler-Toledo S.A.E. Cornellà del Llobregat, Barcelona, España). Argon adsorption–desorption isotherms were measured at 87 K on 3Flex Micromeritics equipment (Malvern Panalytical, Worcestershire, UK); prior, the samples were degassed at 110 °C and high vacuum during 10 h. The specific surface area was calculated by the Brunauer–Emmett–Teller (BET) equation [41]. The pore volume was assessed using the Dubinin–Radushkevich equations. The pore size distribution was estimated using non-local DFT calculations, assuming a kernel model of split pore, Ar-carbon at 87 K.

**Scheme 1:**
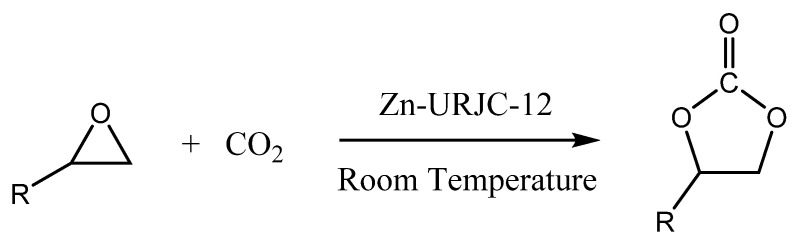
Cycloaddition reaction of CO_2_ and epoxides to carbonates.

Catalytic Studies of Zn-URJC-12 for the Cycloaddition of CO_2_ and Epoxides. In a typical experiment, 1 mmol of epoxide, 1.5 mol% of degassed Zn-URJC-12 catalyst (based on active metal sites), and 0.05 mmol of tetrabutylammonium bromide (TBAB) were added to a stainless-steel autoclave (see Scheme 1). The system was purged with CO_2_ three times before pressurization. The reaction was carried out at room temperature under moderate stirring. Upon completion, residual CO_2_ was carefully released, and the catalyst was recovered by centrifugation. Reaction conversion and selectivity were determined by ^1^H NMR spectroscopy using CDCl_3_ as the solvent and 1,2,4,5-tetrachloro-3-nitrobenzene as the internal standard.

## 3. Results and Discussion

### 3.1. Catalyst Characterization

**Figure 1:**
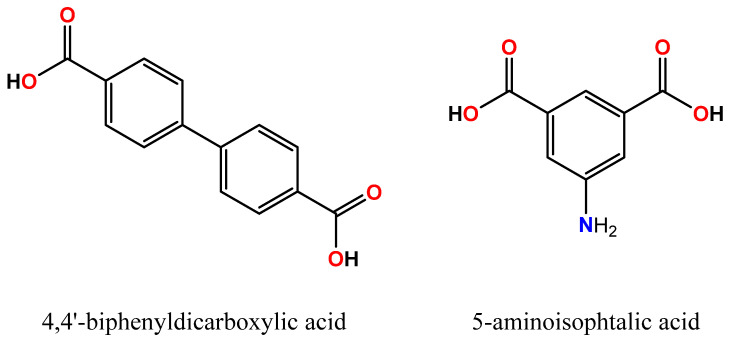
Organic linkers present in Zn-URJC-12 material.

The new material was crystallized at 90 °C using a mixture of DMF and water as solvents. The organic linker 4,4′-biphenyldicarboxylic acid (Figure 1) was selected to increase the pore size of the MOF, thereby reducing steric hindrance caused by bulky epoxides within the cavities of Zn-URJC-12. In parallel, 5-aminoisophthalic acid was incorporated to enhance CO_2_ reactivity due to the presence of amine functional groups, as previously reported [19]. The resulting pink-colored crystals were washed several times with DMF and dried under vacuum for 3 h. The structure of this novel Zn-based MOF was characterized by single-crystal X-ray diffraction.

**Figure 2:**
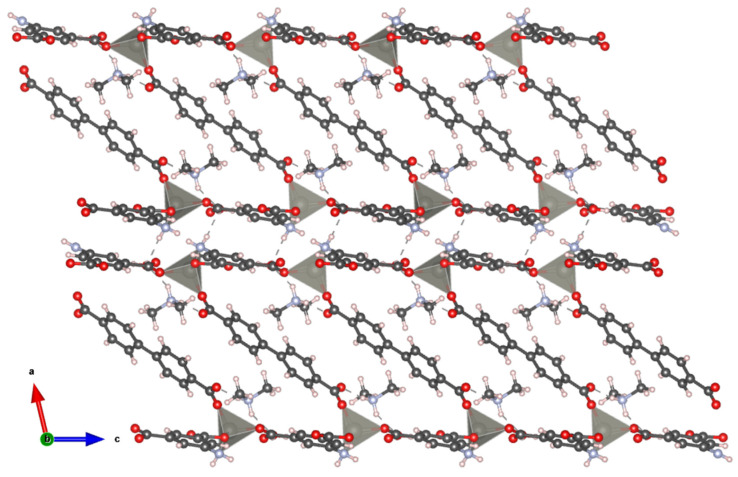
Crystalline structure of Zn-URJC-12 along [010] direction. Zinc, oxygen, nitrogen, carbon, and hydrogen are represented in grey, red, blue, black, and white, respectively. The positional disorder of the biphenyl ligand has been removed for clarity.

Zn-URJC-12 crystallizes in the monoclinic space group C2/c (No. 15), with unit cell parameters *a* = 29.3943 Å, *b* = 8.2366 Å, *c* = 15.8012 Å, and β = 103.342°, yielding a total unit cell volume of 3722.4 Å^3^ (Table S1.1). The framework, with the formula {(C_2_NH_6_)_2_[Zn(C_14_H_8_O_4_)(C_8_H_5_NO_4_)_2_]·2H_2_O}_n_ or {(DMA)_2_[Zn_2_(bpdc)(5-aip)_2_]·2H_2_O}_n_ (where DMA = dimethylammonium, bpdc = 4,4′-biphenyldicarboxylate, and 5-aip = 5-aminoisophthalate), consists of anionic [Zn**_2_**(bpdc)(5-aip)_2_] layers extending along the *bc* plane. These layers are separated by DMA cations and water molecules, which interact via hydrogen bonding (Figure 2 and Figure S1.1). The layers themselves are composed of [Zn(5-aip)] sublayers interconnected by bpdc linkers. Each Zn(II) center adopts a distorted tetrahedral coordination geometry (ZnO_3_N), with Zn–O bond lengths ranging from 1.93 to 1.99 Å and a Zn–N bond length of 2.05 Å (Figure S1.2; Tables S1.2 and S1.3). Each Zn atom is coordinated by one bpdc and three 5-aip ligands, each originating from a different molecule. Regarding ligand coordination, each 5-aip ligand bridges three Zn atoms through two monodentate carboxylate oxygen atoms (one from each carboxyl group) and the amino group. Each functional group also forms hydrogen bonds with distinct water molecules (Figure S1.3a). The bpdc linker connects two Zn atoms via monodentate coordination through one oxygen atom of each carboxylate group, while the second oxygen atoms engage in hydrogen bonding with DMA cations (Figure S1.3b). According to PLATON calculations, water molecules occupy approximately 7% of the unit cell volume and interact with both inorganic layers. In contrast, DMA cations interact primarily with the carboxylate groups of one layer and with water molecules.

**Figure 3:**
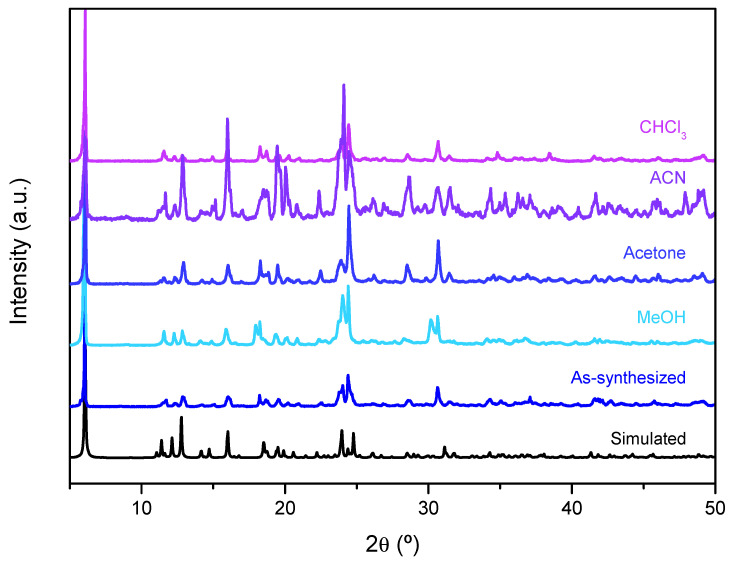
XRD of simulated and experimental patterns of Zn-URJC-12.

The X-ray diffraction (XRD) pattern of the as-synthesized material confirmed the successful formation of the Zn-URJC-12 framework, as the experimental pattern matched the simulated one, displaying characteristic diffraction peaks at 6.18°, 11.51°, 12.27°, 12.86°, and 15.93° (Figure 3). Additionally, the chemical stability of the material was evaluated in various organic solvents. Notably, Zn-URJC-12 exhibited excellent stability in methanol (MeOH), acetone, acetonitrile, and CHCl_3_, as evidenced by the preservation of its most representative diffraction peaks at the same 2θ values in the PXRD pattern (Figure 3).

**Figure 4:**
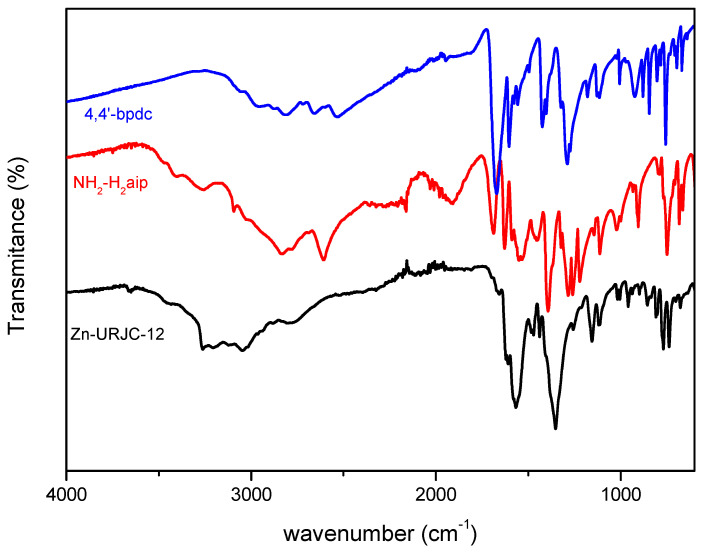
FT-IR of Zn-URJC-12, NH_2_-H_2_aip, and 4,4′-bypdc.

The FT-IR spectrum of Zn-URJC-12 (Figure 4) displays characteristic vibrational bands corresponding to functional groups present in the framework. Especially, bands in the range of 3260–3047 cm^−1^ are attributed to N–H stretching vibrations from both the amine and dimethylammonium groups. The broad shape of the ν(N–H) band is indicative of coordination between the –NH_2_ group and the metal center, as previously reported in the literature [42,43,44]. Additionally, the stretching vibrations of the carboxylate groups (ν(C=O)) are observed at 1602 and 1567 cm^−1^.

**Figure 5:**
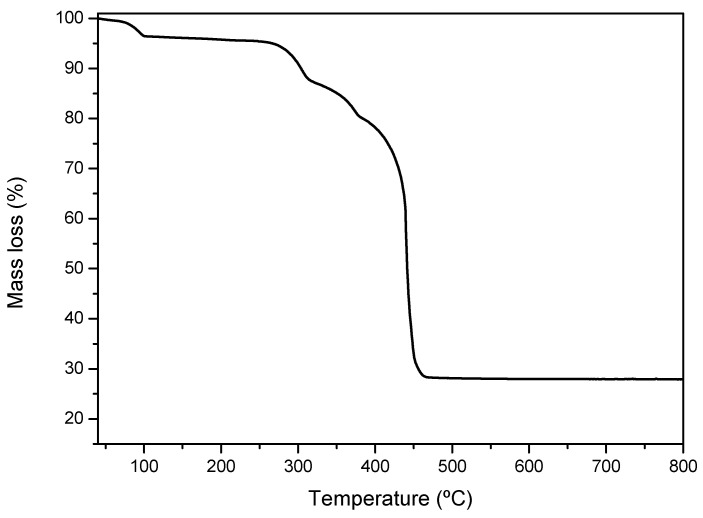
Thermogravimetric analysis of Zn-URJC-12 material.

The thermal stability of Zn-URJC-12 was evaluated by thermogravimetric analysis (TGA) under an air atmosphere (Figure 5). The TGA curve revealed four distinct mass loss events. The first mass loss, occurring around 100 °C, corresponds to the release of water molecules retained within the pores of the material. The subsequent mass losses, observed between 280 °C and 450 °C, are attributed to the stepwise decomposition of the organic linkers, ultimately leading to the collapse of the MOF structure.

**Figure 6:**
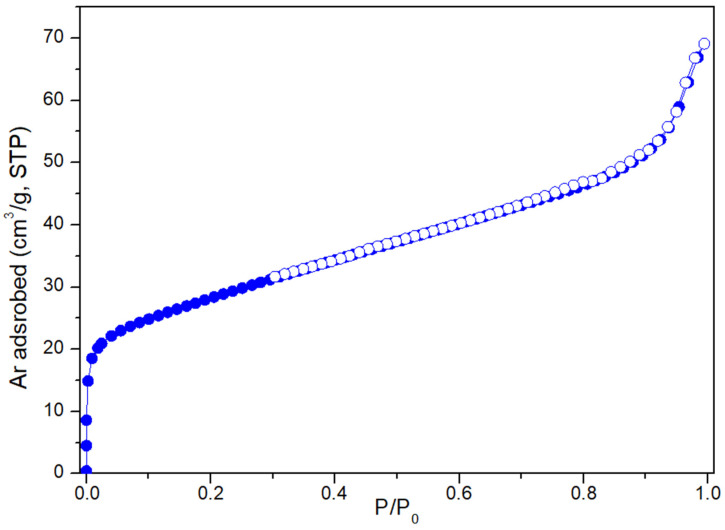
Ar adsorption/desorption isotherm of Zn-URJC-12.

Following the TGA analysis, the Zn-URJC-12 material was immersed in methanol for three days to exchange residual water and DMF molecules retained within its pores. Subsequently, the material was degassed at 110 °C under vacuum for 10 h. Argon adsorption–desorption isotherms were then recorded at 87 K. As shown in Figure 6, the isotherm corresponds to a microporous material, with a BET surface area of 85 m^2^/g, a total pore volume of 0.082 cm^3^/g, and a pore size distribution centered at 5.3 and 9.9 Å (Figure S2.1).

### 3.2. Catalytic Studies of Zn-URJC-12 for the Cycloaddition of CO_2_ and Epoxides

The cycloaddition reaction of CO_2_ with various epoxides was carried out in a 100 mL stainless-steel autoclave under the following optimized conditions: 1.5 mol% of Zn-URJC-12 catalyst, 5 mol% of tetrabutylammonium bromide (TBAB), 12 bar of CO_2_, and room temperature for 24 h. These conditions were previously identified as optimal in our research group using a different Zn-based MOF material [20].

To evaluate the influence of different substituents on the epoxide ring, four substrates were tested: propylene oxide, epichlorohydrin, allyl glycidyl ether, and styrene oxide. The catalytic performance of Zn-URJC-12 with each substrate is summarized in Table 1.

As shown in the results, the use of propylene oxide as a substrate led to a reaction yield of 100%, attributed to its small molecular size, which facilitates diffusion into the pores of the catalyst. In the case of epichlorohydrin, a conversion of 90% was achieved, as this epoxide remains relatively compact, despite the presence of a chlorine atom in its structure. However, when allyl glycidyl ether was used, the conversion decreased due to the longer substituent chain, which increases steric hindrance within the pores and limits the interaction between the substrate and the active metal centers.

Styrene oxide, which contains a bulky aromatic ring, exhibited the lowest conversion (59%), likely due to the significant steric hindrance imposed by the aromatic group within the MOF’s pores, which have diameters of 5.3 and 9.9 Å, as determined by Ar adsorption–desorption isotherms. Additionally, the molecular dimensions of styrene oxide have been estimated at approximately 7.2 × 4.2 Å [45], which would further hinder its diffusion into the cavities of Zn-URJC-12. Furthermore, the selectivity toward cyclic carbonate in the reaction with styrene oxide was slightly below 99% due to the formation of a side product, 2,5-diphenyl-1,4-dioxane (Figure S3.5), as previously reported in the literature [6,19,21].

In addition, the catalytic performance of Zn-URJC-12 was compared with other common zinc-based catalysts, including zinc nitrate, zinc bromide, and the Zn-based MOF ZIF-8, in the cycloaddition reaction between CO_2_ and epichlorohydrin. The results are summarized in Table 2. As shown, Zn-URJC-12 exhibited the highest epichlorohydrin conversion, which is attributed to the presence of –NH_2_ groups from the 5-aminoisophthalic acid linker. These groups can interact with CO_2_ molecules, enhancing their activation and reactivity.

Zinc nitrate achieved a conversion of 80%, outperforming zinc bromide, likely due to its homogeneous catalytic behavior. In contrast, ZIF-8 showed a lower conversion (76%) compared to Zn-URJC-12 (90%), despite also containing nitrogen atoms in its organic linker. This difference is attributed to the lower basicity of the nitrogen atoms in ZIF-8 relative to those in Zn-URJC-12, which limits their ability to activate CO_2_ [19]. The variation in nitrogen basicity between the two MOFs is a key factor responsible for the observed differences in catalytic performance.

Furthermore, the catalytic performance of Zn-URJC-12 was compared with other MOF materials previously reported in the literature. As shown in Table 3, achieving reaction yields above 80% typically requires reaction temperatures exceeding 80 °C. In some cases, even under such conditions, the catalytic results were not particularly promising. These findings underscore the importance of promoting both the epoxide ring-opening and the activation of the CO_2_ molecule. This activation is not only enhanced by elevated temperatures and CO_2_ pressures but also by a synergistic interaction between the metal center and the functional groups of the organic linker [46].

**Figure 7:**
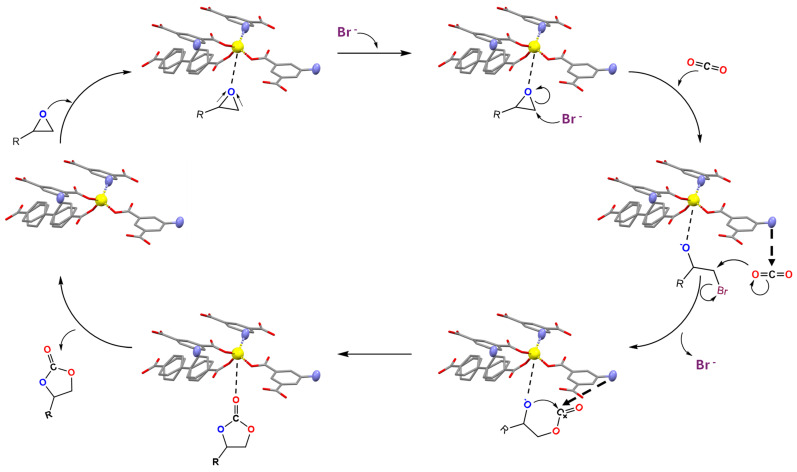
Proposed reaction mechanism of cycloaddition reaction between CO_2_ and epoxides catalyzed by Zn-URJC-12.

The proposed reaction mechanism is illustrated in Figure 7. In the first step, the epoxide coordinates to the zinc center through its oxygen atom, inducing polarization of the C–O bonds. This activation facilitates a nucleophilic attack by the bromide ion from TBAB on the less substituted carbon atom of the epoxide, resulting in ring opening. In the second step, the amine group from the 5-aminoisophthalic acid linker interacts with the CO_2_ molecule, promoting a nucleophilic attack by one of the oxygen atoms of CO_2_ on the carbon atom bearing the bromide. This leads to the displacement of the bromide ion in the third step. Finally, the oxygen atom from the opened epoxide attacks the carbon atom of the CO_2_ moiety, closing the ring to form the cyclic carbonate and regenerating the catalyst.

**Figure 8:**
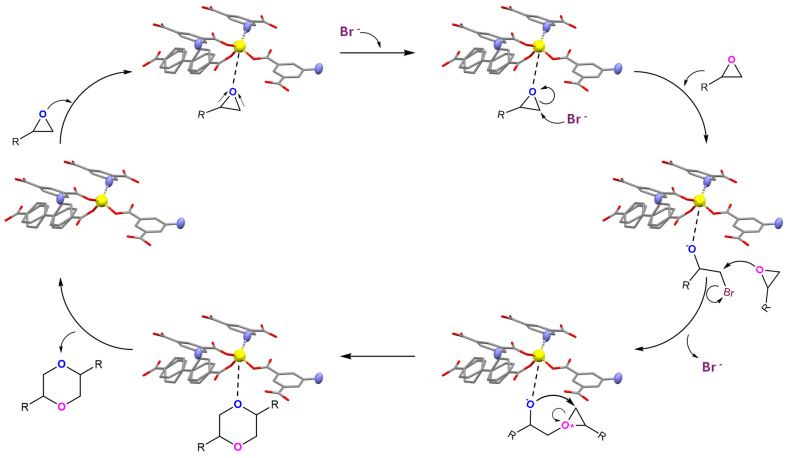
Proposed reaction mechanism of cyclodimerization catalyzed by Zn-URJC-12.

In addition to the formation of the cyclic carbonate, when a bulky epoxide such as styrene oxide is used, a secondary cyclodimerization reaction may occur, leading to the formation of dioxanes. Therefore, the mechanism of this secondary reaction is proposed and illustrated in Figure 8. This pathway initially mirrors the early stages of the cycloaddition reaction mechanism; however, the critical distinction lies in the nucleophilic attack. Rather than involving a nucleophilic attack by one of the oxygen atoms of the CO_2_ molecule, the nucleophile in this case is the oxygen atom of a second epoxide molecule. This atom attacks the carbon atom bonded to the oxygen of the reaction intermediate. Subsequently, the oxygen atom within the intermediate undergoes an intramolecular nucleophilic attack on the less substituted carbon of the second epoxide, resulting in ring closure and the release of a by-product into the reaction medium.

**Figure 9:**
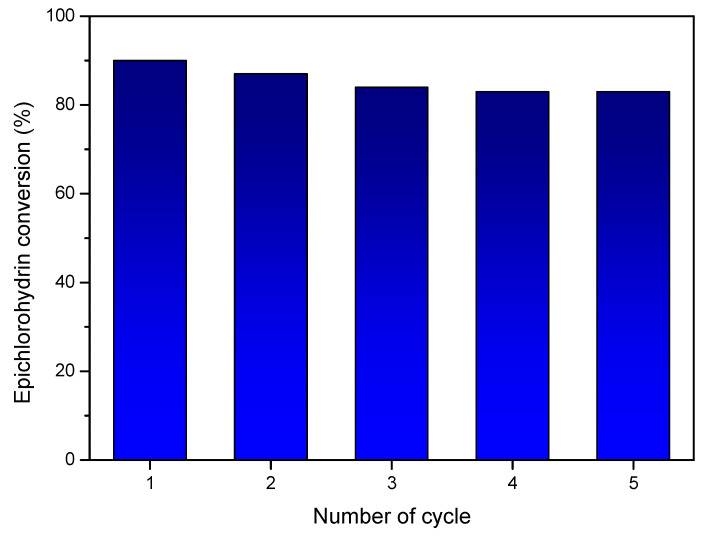
Recyclability of Zn-URJC-12 material for five consecutive cycles.

Finally, the recyclability of the Zn-URJC-12 material was evaluated over five consecutive cycles using epichlorohydrin as the substrate under identical reaction conditions. As shown in Figure 9, the epoxide conversion decreased from 90% to 83% over the course of the cycles. This decline in epichlorohydrin conversion is attributed to the partial retention of the product within the material’s cavities, likely due to hydrogen bonding between the hydrogen atoms of the –NH_2_ groups and the oxygen atom of the carbonate. The washing steps between cycles were insufficient to completely remove the cyclic carbonate retained within the structure. After the final reaction cycle, the material was characterized by XRD, revealing diffraction patterns identical to those of the as-synthesized material. These results confirm that Zn-URJC-12 remains structurally stable and catalytically active for the cycloaddition reaction between CO_2_ and epoxides (Figure S4.6).

## 4. Conclusions

The synthesis and characterization of the novel Zn-URJC-12 material have been reported. This new Zn-based metal–organic framework exhibits a two-dimensional layered structure and is constructed from two distinct organic ligands, 4,4′-biphenyldicarboxylic acid and 5-aminoisophthalic acid, incorporating Lewis basic groups within its framework. The –NH_2_ groups are coordinated to zinc ions, as confirmed by single-crystal X-ray diffraction and infrared spectroscopy, which show a broad absorption band in the range of 3260–3047 cm^−1^. Thermogravimetric analysis revealed that Zn-URJC-12 is thermally stable up to 250 °C, with a significant weight loss occurring between 250 and 450 °C, corresponding to structural decomposition. The material exhibits a BET surface area of 85 m^2^/g and a pore volume of 0.082 cm^3^/g. Zn-URJC-12 demonstrated excellent catalytic performance in the cycloaddition reaction of CO_2_ with epoxides under mild conditions (1.5 mol% catalyst, 5 mol% co-catalyst, 12 bar CO_2_, 24 h, room temperature). The reaction yields for propylene oxide, epichlorohydrin, allyl glycidyl ether, and styrene oxide were 100, 91, 76, and 59%, respectively, indicating that steric hindrance within the MOF cavities depends on the size of the epoxide substituent. Finally, the material maintained its catalytic activity over five consecutive cycles using epichlorohydrin as the substrate, confirming that Zn-URJC-12 is a highly promising heterogeneous catalyst for the cycloaddition of CO_2_ and epoxides.

## Figures and Tables

**Table 1:** Reaction results of cycloaddition reaction between CO_2_ and epoxides.

Epoxide	Cyclic Carbonate	Conversion (%) ^a^	Selectivity (%) ^b^
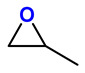	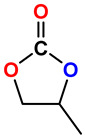	100	>99
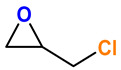	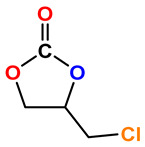	91	>99
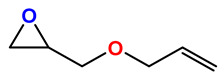	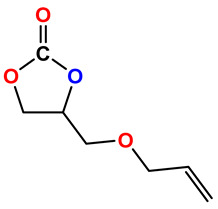	76	>99
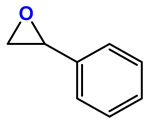	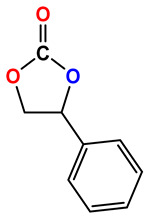	59	64

^a,b^ The conversion and selectivity of the cycloaddition reaction were determined by H-RMN with CDCl_3_ as the solvent.

**Table 2:** Reaction results obtained with other Zn-based materials ^a,b^.

Material	Conversion (%) ^a^	Selectivity (%) ^b^
Zn-URJC-12	90	>99
Zn(NO_3_)_2_	80	>99
ZnBr_2_	33	>99
ZIF-8	76	95

^a,b^ The conversion and selectivity of the cycloaddition reaction were calculated by H-RMN using CDCl_3_ as solvent.

**Table 3:** Comparison with different MOFs published in the literature.

MOF	Pressure (Bar)	Temperature (°C)	Co-Catalyst/Time (h)	Yield (%)	Ref
Zn-URJC-12	12	R.T.	TBAB/24	90	This work
Cu-MOF-1	1	R.T.	TBAB/48	88	[47]
ZnTCPP	1	140	-/14	87	[48]
ZIF-8	7	80	-/4	44	[49]
NR_2_-ZIF-8	7	80	-/4	73	
Ni(salphen)-MOF	20	80	TBAB/4	84	[50]
F-IRMOF-3	20	140	-/15	80	[51]
HKUST-1	7	100	-/4	34	[52]
Zn-MOF	1	70	TBAB/24	98	[53]
Zn-MOF	1	40	TBAB/2	100	[54]
ZnCo-ZIF	7	100	-/4	99	[55]
HE-ZIF	10	100	-/8	99	[56]

## Data Availability

The authors confirm that the data supporting the findings of this study are available within the article.

## Supplementary Materials

The supporting information can be downloaded at: https://www.mdpi.com/article/10.3390/nano15131018/s1. S1. Crystallographic data of Zn-URJC-12 and additional figures of the structure. S2. Characterization of Zn-URJC-12. S3. Catalytic results and characterization from reactions with epoxides. S4. Recyclability of Zn-URJC-12.
