# Supplementary material for: Zn-URJC-12 Material Constituted of Two Different Organic Ligands for CO2 Valorization into Cyclic Carbonates
Source: Nanomaterials (Basel). 2025 Jul 1;15(13):1018. doi: 10.3390/nano15131018 (PMC12250655; doi:10.3390/nano15131018)
Supplement: Supplementary file 1 [file nanomaterials-15-01018-s001.zip › nanomaterials-3702940-supplementary.pdf]

Zn-URJC-12 Material Constituted of Two Different Organic Ligands for CO_2 Valorization into Cyclic Carbonates

Jesús Tapiador [1], Pedro Leo [1], Pablo Salcedo-Abraira [2], Antonio Rodríguez-Diéguez [2] and Gisela Orcajo [1]

[1]Chemical and Environmental Engineering Group, Escuela Superior de Ciencias Experimentales y Tecnología (ESCET), Universidad Rey Juan Carlos, c/Tulipán s/n, 28933 Móstoles, Spain.
[2]Department of Inorganic Chemistry, University of Granada, Avda. Fuentenueva s/n, 18071 Granada, Spain.

S1. Crystallographic data of Zn-URJC-12 and additional figures of the structure.

S2. Characterization of Zn-URJC-12.

S3. Catalytic results and characterization from reactions with epoxides.

S4. Recyclability of Zn-URJC-12.

S1. Crystallographic data of Zn-URJC-12 and additional figures of the structure.

Table S1. 1. Crystal data and structure refinement for Zn-URJC-12.

**Identification code**	**Zn-URJC-12**
CCDC number	2449688
Empirical formula	$C_{34}H_{38}N_4O_{14}Zn_2$
Formula weight	857.42
Temperature/K	100.00
Crystal system	monoclinic
Space group	*C*2/*c*
a/Å	29.3943(1)
b/Å	8.2366(3)
c/Å	15.8012(7)
α/°	90
β/°	103.342(2)
γ/°	90
Volume/Å^3	3722.4(3)
Z	4
ρ_{calc}g/cm^3	1.530
μ/mm^{-1}	1.361
F(000)	1768.0
Crystal size/mm^3	0.1 × 0.09 × 0.04
Radiation	MoK*α* (*λ* = 0.71073)
2Θ range for data collection/°	5.146 to 57.376
Index ranges	-39 ≤ h ≤ 39,<br>-11 ≤ k ≤ 10,<br>-20 ≤ l ≤ 21
Reflections collected	18150
Independent reflections	4766 [R_{int} = 0.0718, R_{sigma} = 0.0582]
Data/restraints/parameters	4766/16/277
Goodness-of-fit on F^2	1.077
Final R indexes [I>=2σ (I)]	R_1 = 0.0543, wR_2 = 0.1244
Final R indexes [all data]	R_1 = 0.0869, wR_2 = 0.1579
Largest diff. peak/hole / e Å^{-3}	1.43/-0.91

Table S1. 2. Selected bond angles for Zn-URJC-12.

**Atom**	**Atom**	**Atom**	**Angle/˚**
O5^1	Zn1	O3	92.02(11)
O5^1	Zn1	N1^2	116.42(12)
O3	Zn1	N1^2	114.79(12)
O1	Zn1	O5^1	111.46(13)
O1	Zn1	O3	117.28(12)
O1	Zn1	N1^2	105.08(13)

$^{1.}$+X,1-Y,-1/2+Z; ^{2}+X,-1+Y,+Z

Table S1. 3. Selected bond lengths for Zn-URJC-12.

Atom	Atom	Length/Å
Zn1	O5[1]	1.966(3)
Zn1	O3	1.996(3)
Zn1	O1	1.931(3)
Zn1	N1[2]	2.048(3)

[1]+X,1-Y,-1/2+Z; [2]+X,-1+Y,+Z

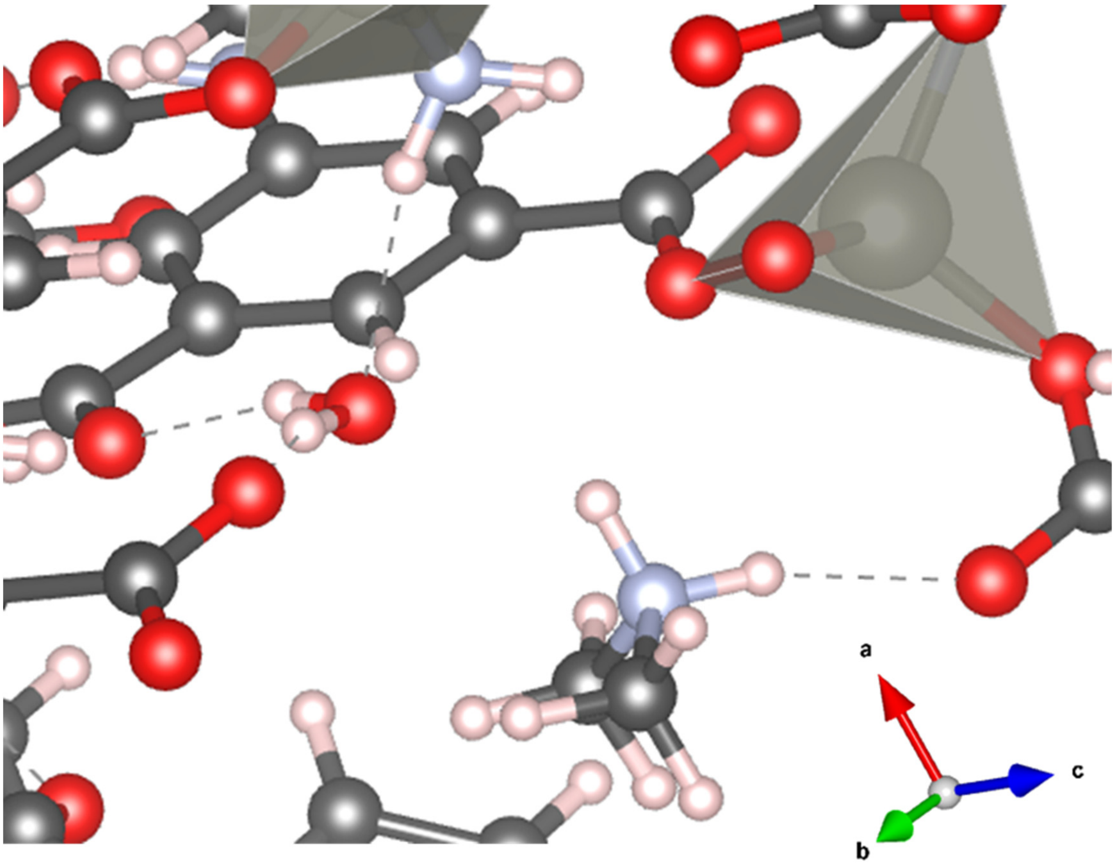

Figure S1. 1. Detailed view of the H-bond interactions in the Zn-URJC-12 structure. Zinc, oxygen, nitrogen, carbon and hydrogen are represented in grey, red, blue, black and white, respectively. .

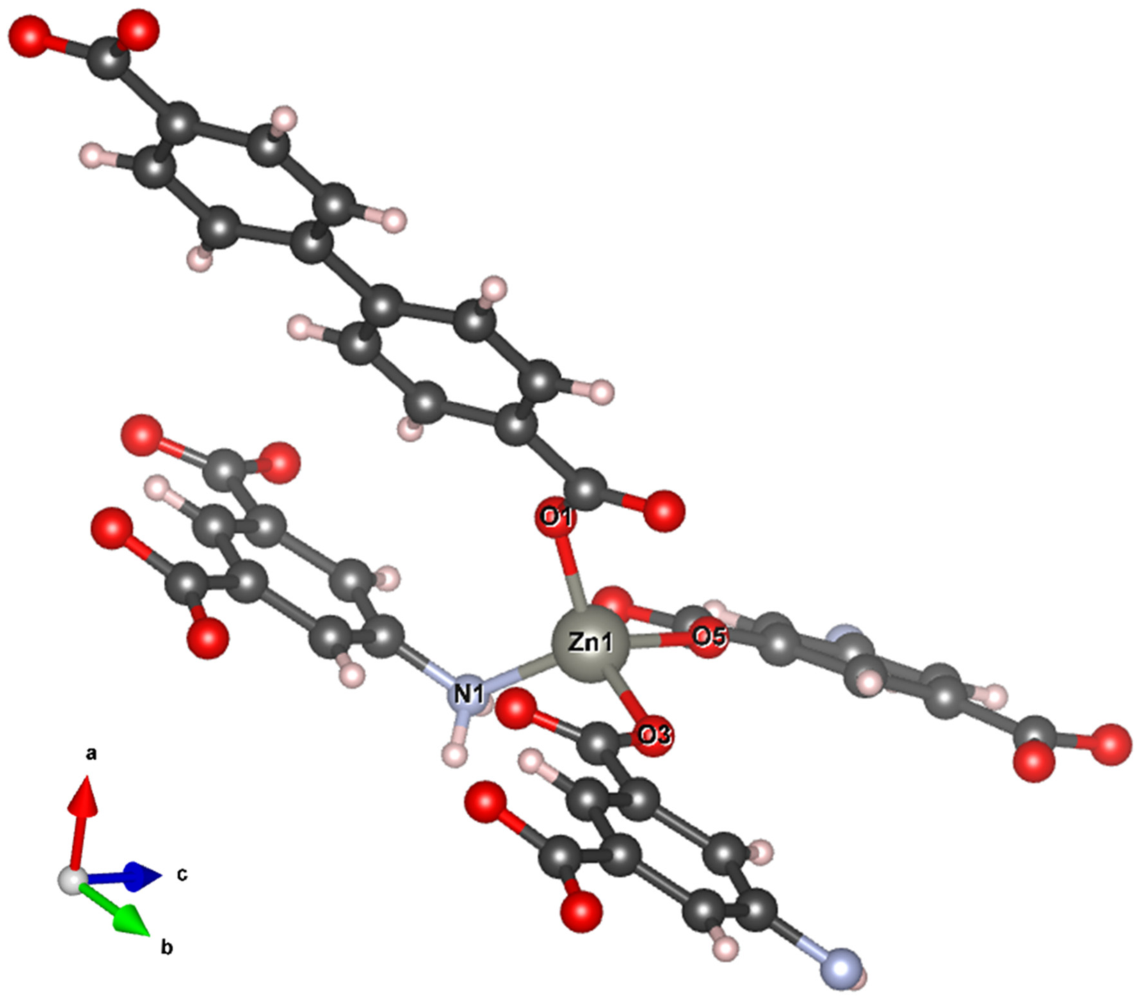

Figure S1. 2. ZnO_3N tetrahedra coordination of Zn-URJC-12. Zinc, oxygen, nitrogen, carbon and hydrogen are represented in grey, red, blue, black and white, respectively. The positional disorder of the biphenyl ligand has been removed in terms of clarity.

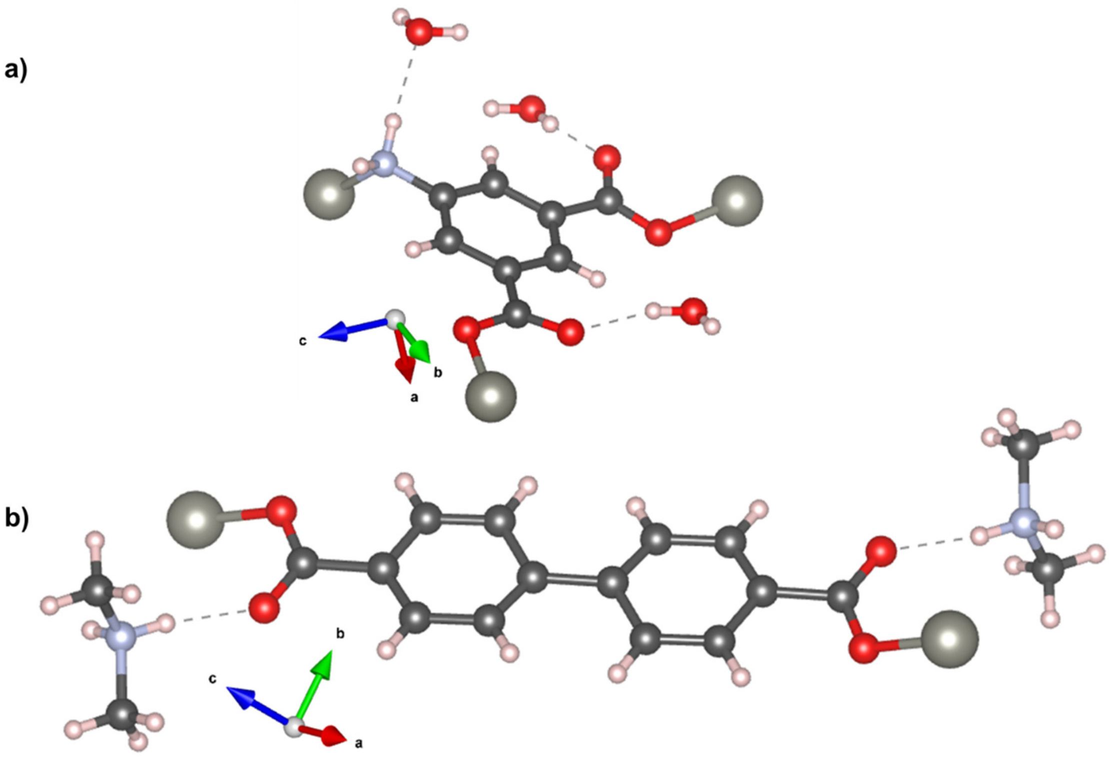

Figure S1. 3. 5-amino isophthalate (a) and 4,4'-biphenildicarboxylate (b) ligands coordination modes and H-bond interactions. Zinc, oxygen, nitrogen, carbon and hydrogen are represented in grey, red, blue, black and white, respectively. The positional disorder of the biphenyl ligand has been removed in terms of clarity.

S2. Characterization of Zn-URJC-12.

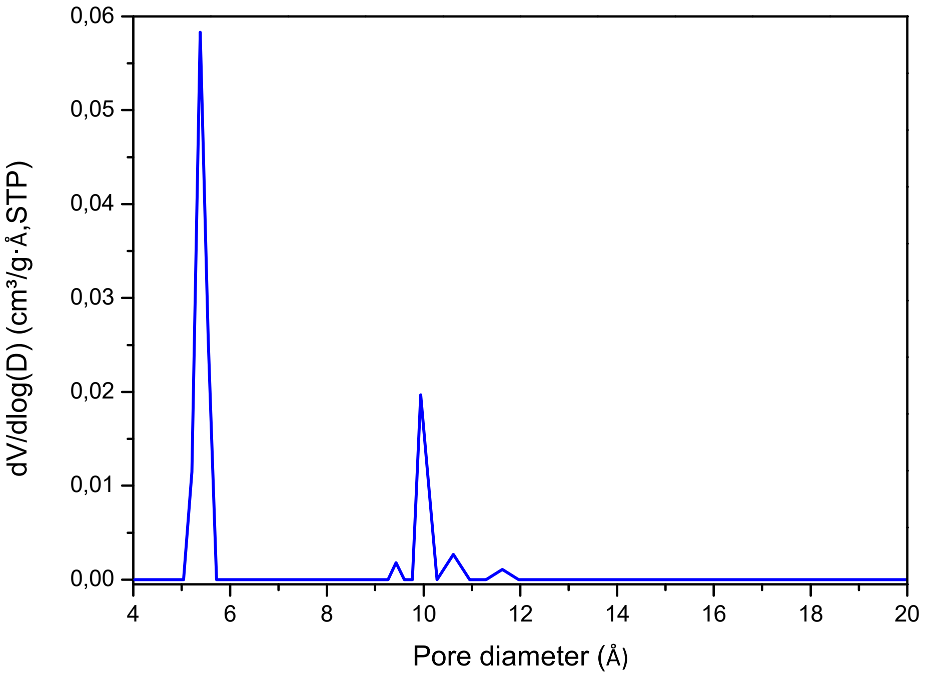

Figure S2. 1 Pore diameter distribution of Zn-URJC-12.

S3. Catalytic results and characterization from reactions with epoxides.

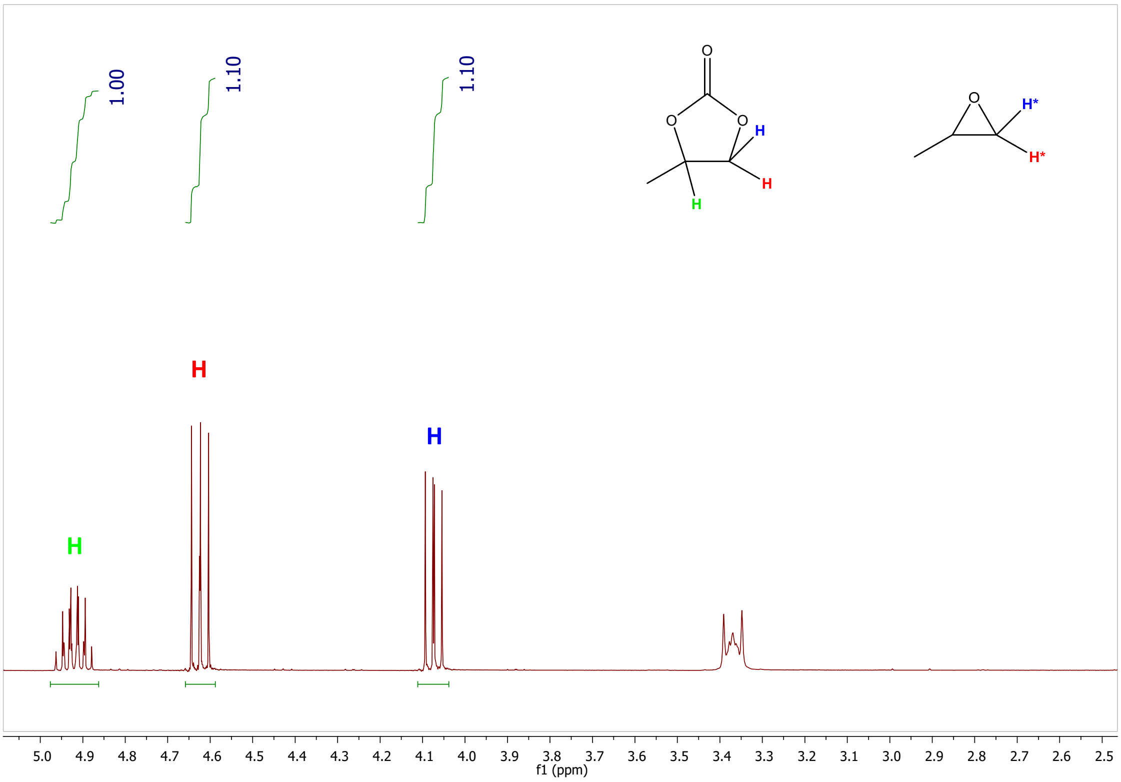

Figure S3. 1. H-NMR of reaction between propylene oxide and CO_2 catalyzed by Zn-URJC-12.

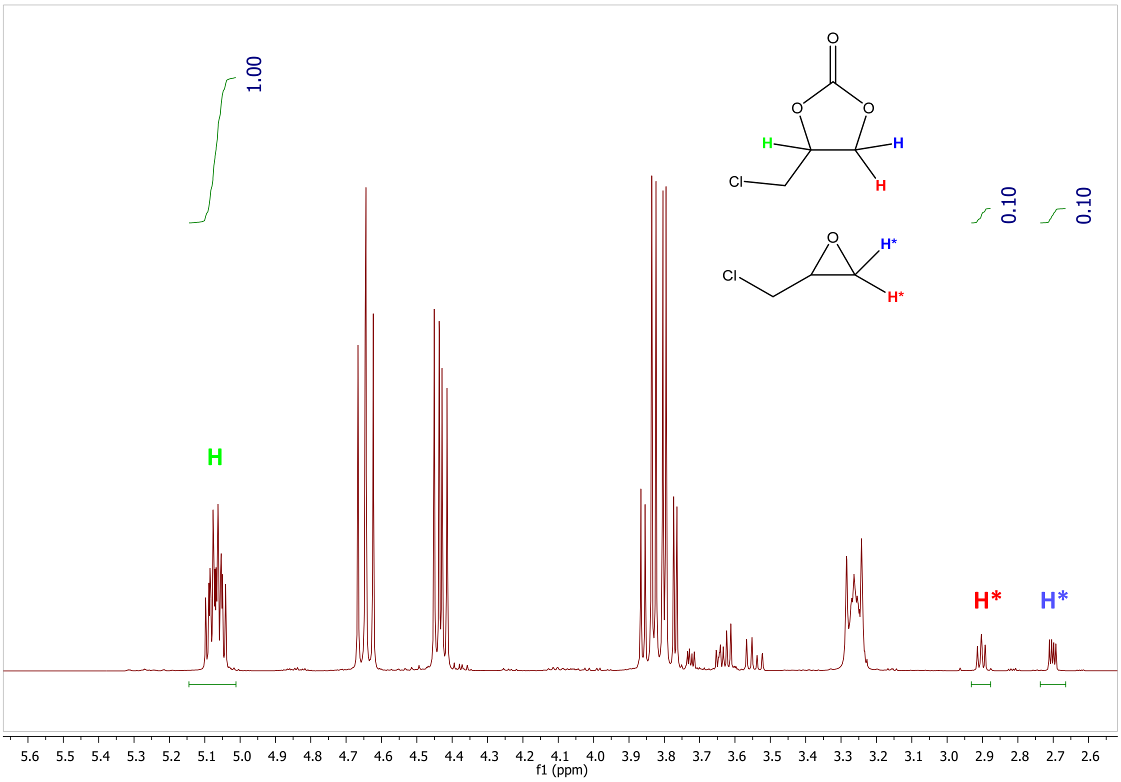

Figure S3. 2. H-NMR of reaction between epichlorohydrin and CO_2 catalyzed by Zn-URJC-12.

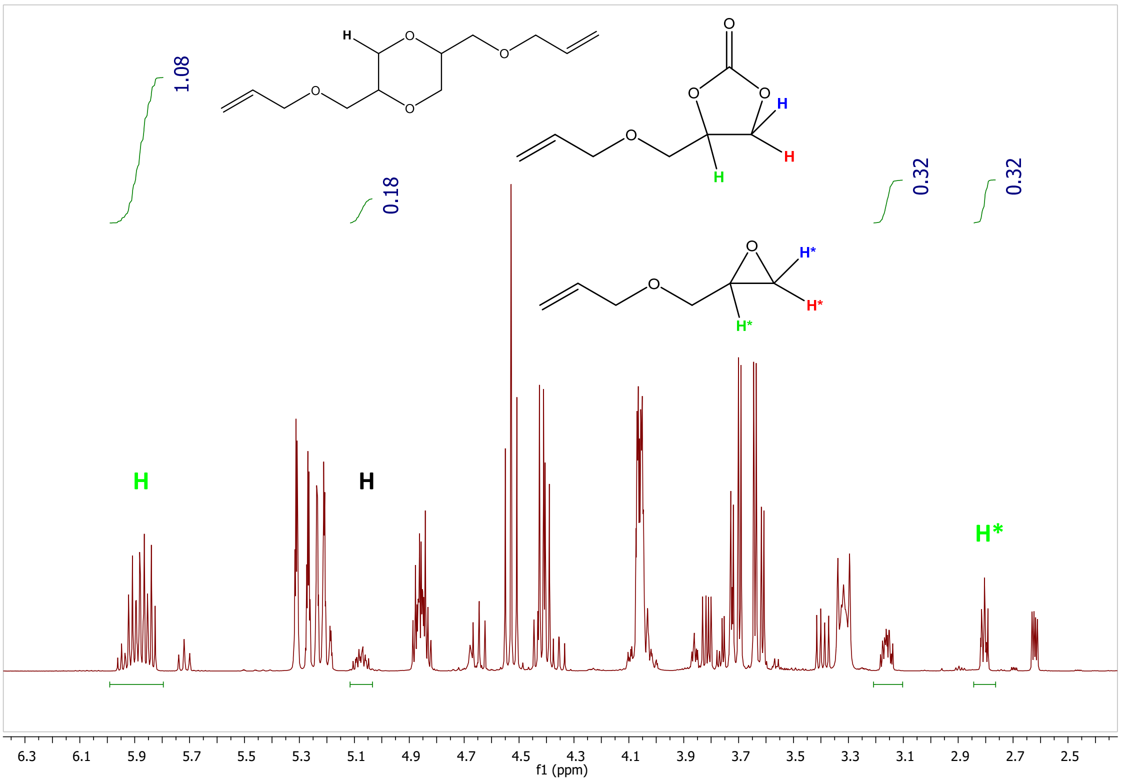

Figure S3. 3. H-NMR of reaction between allyl glycidyl ether and CO_2 catalyzed by Zn-URJC-12.

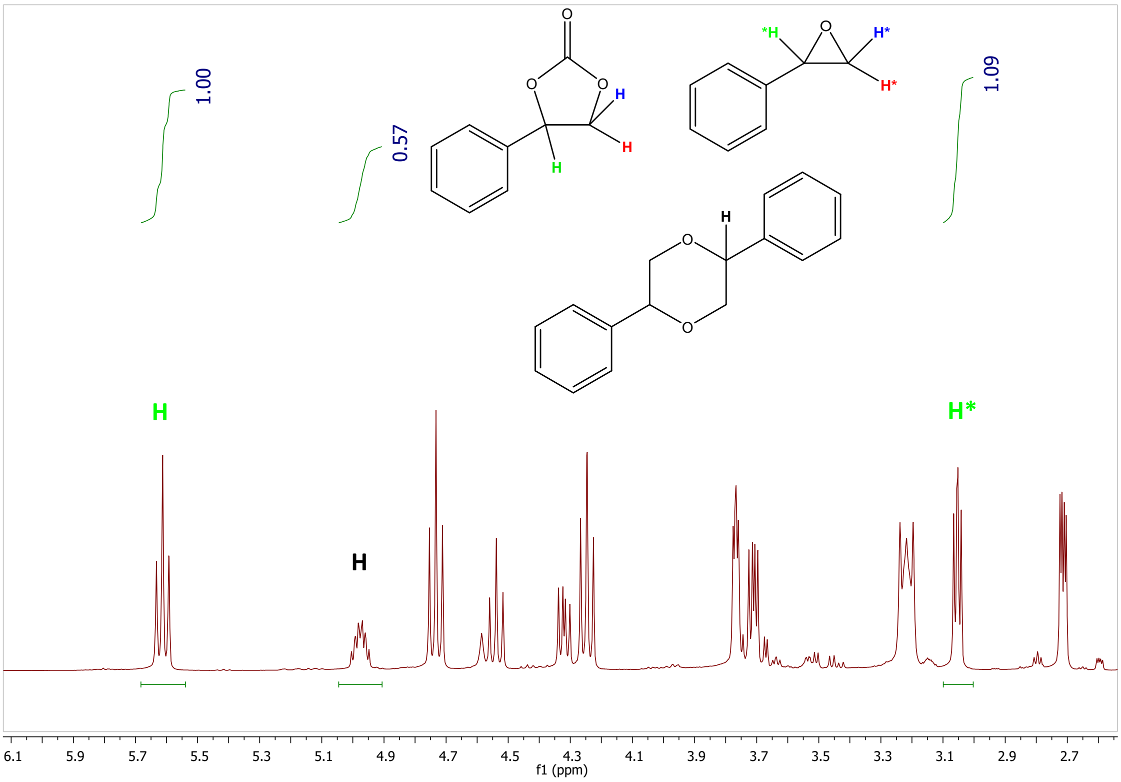

Figure S3. 4. H-NMR of reaction between styrene oxide and CO_2 catalyzed by Zn-URJC-12.

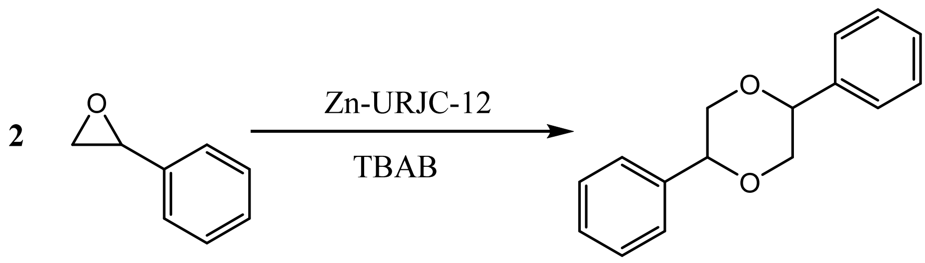

Figure S3. 5. Secondary reaction to produces 2,5-diphenyl-1,4-dioxane.

S4. Recyclability of Zn-URJC-12.

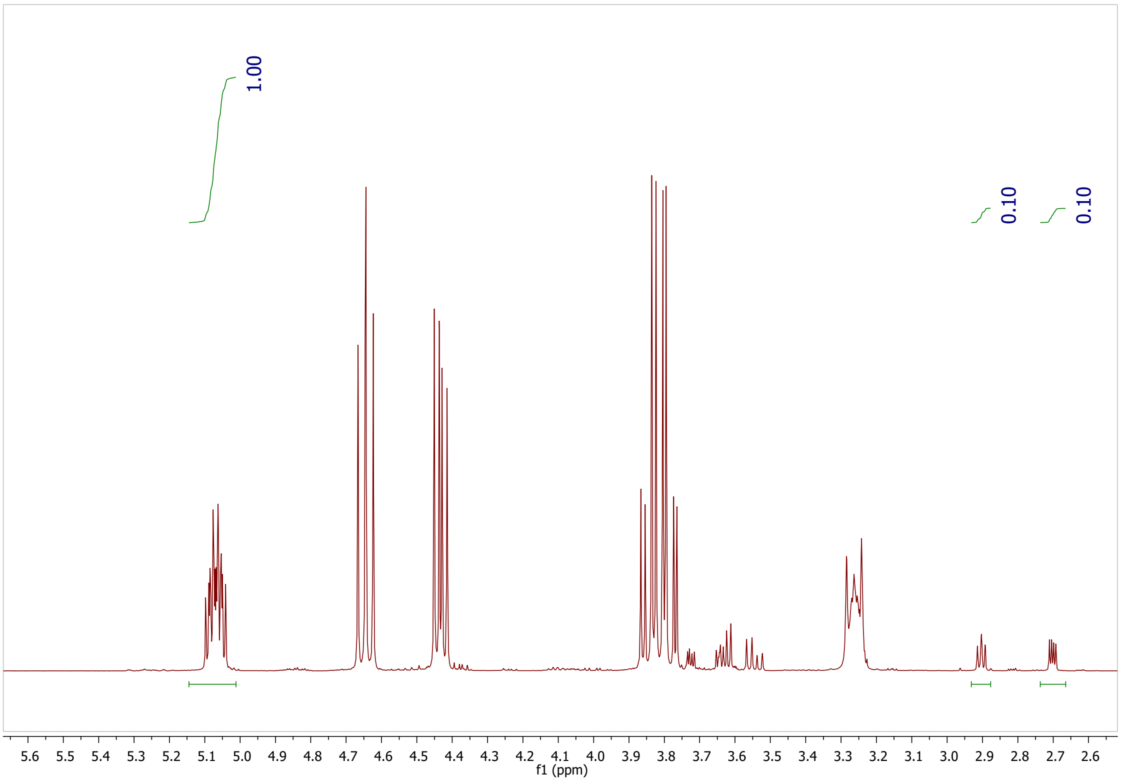

Figure S4. 1. H-NMR of cycle number 1.

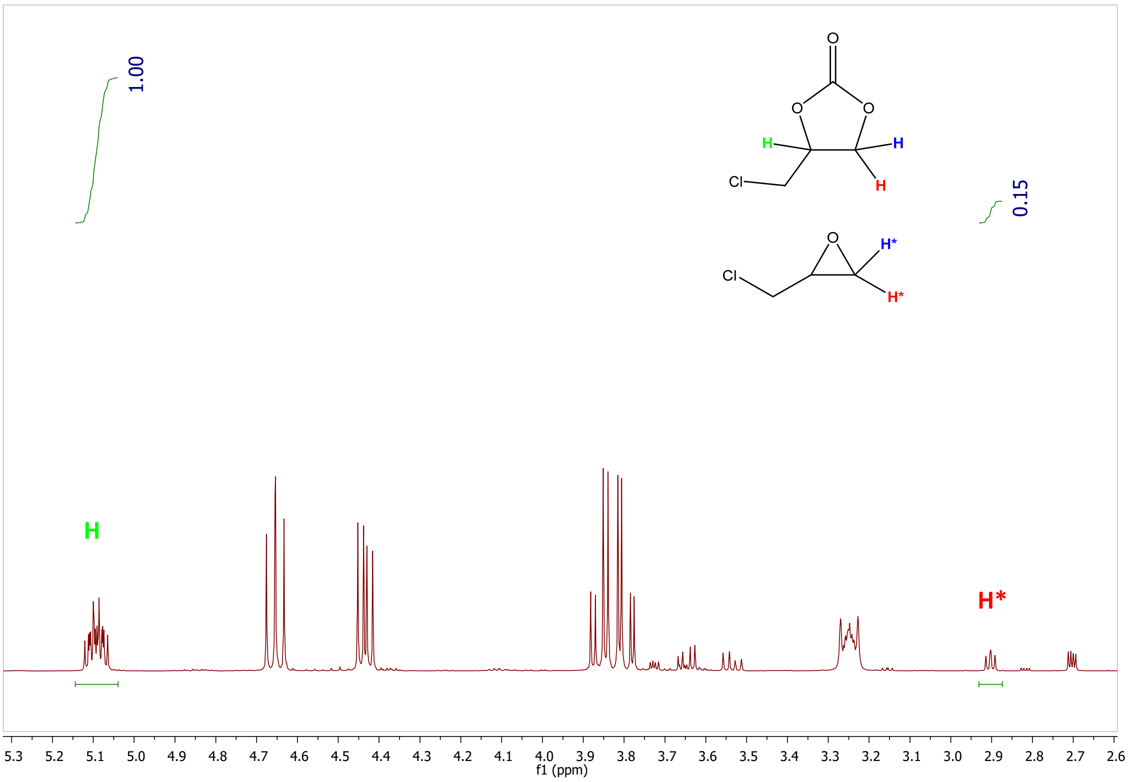

Figure S4. 2. H-NMR of cycle number 2.

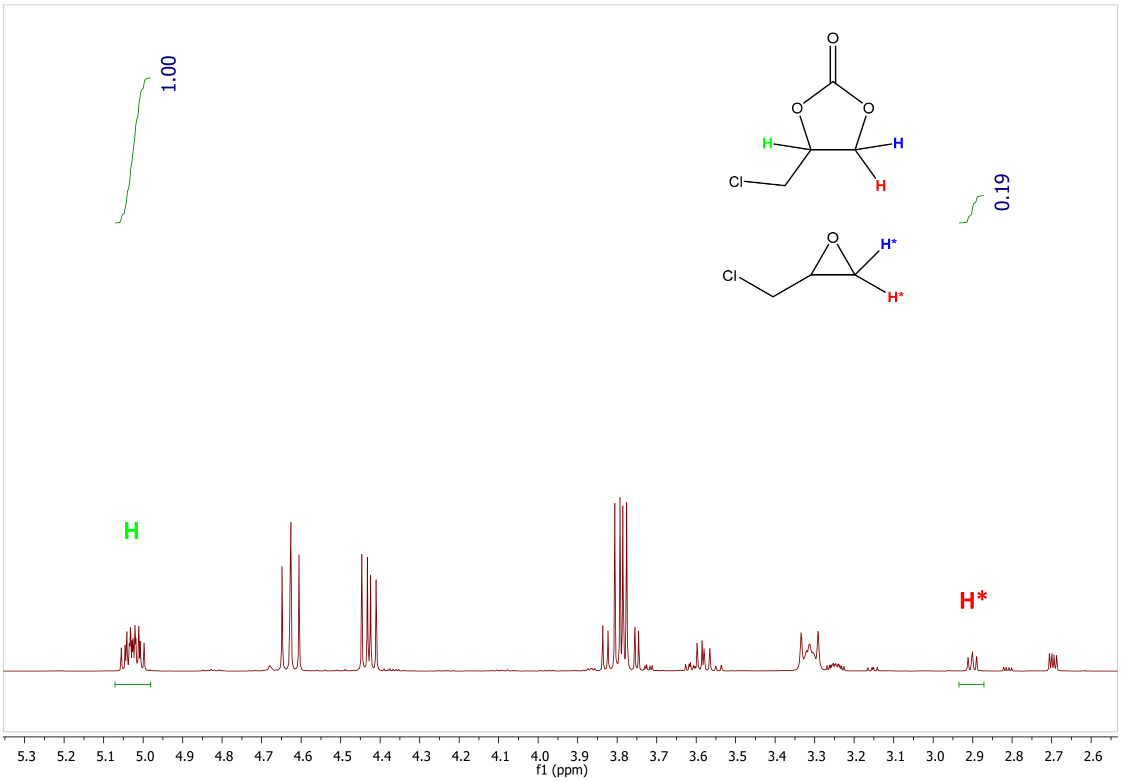

Figure S4. 3. H-NMR of cycle number 3.

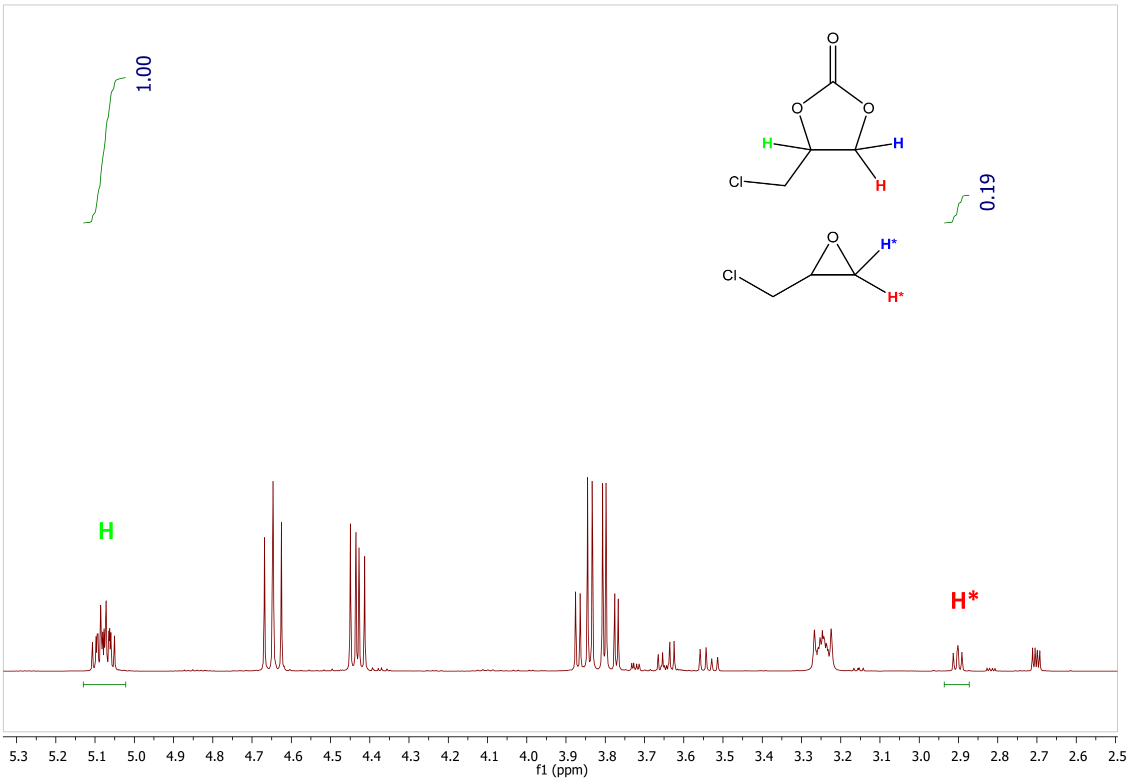

Figure S4. 4. H-NMR of cycle number 4.

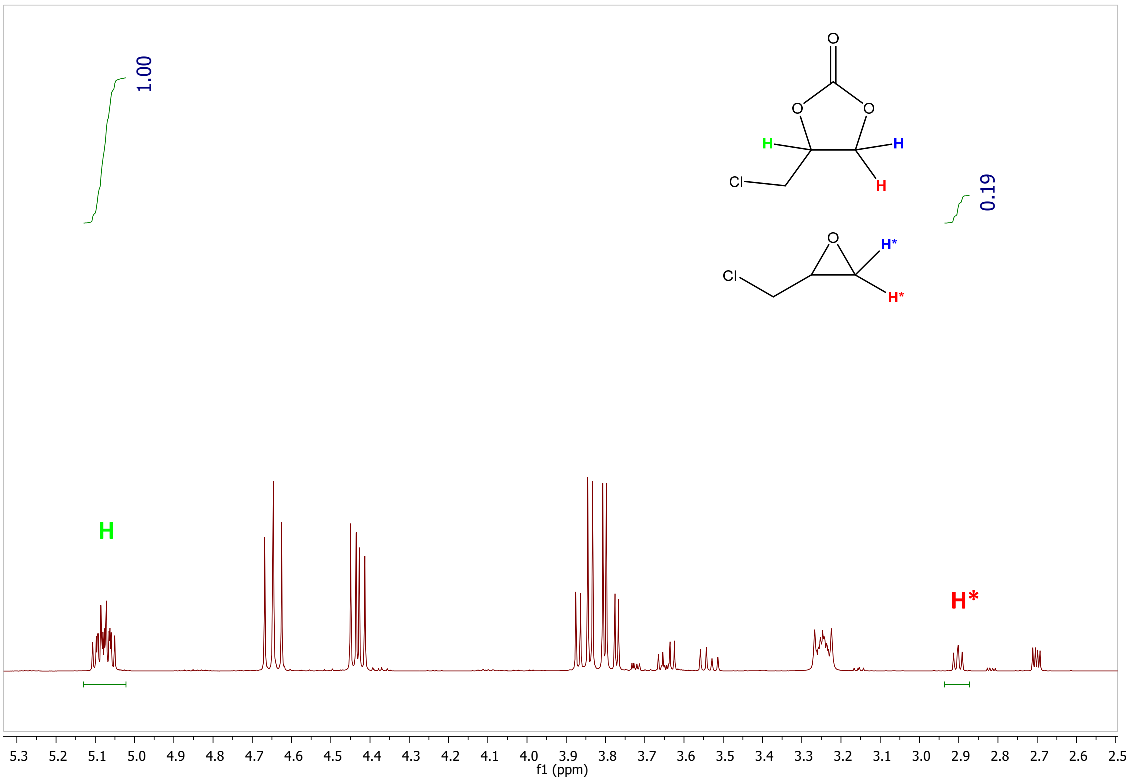

Figure S4. 5. H-NMR of cycle number 5.

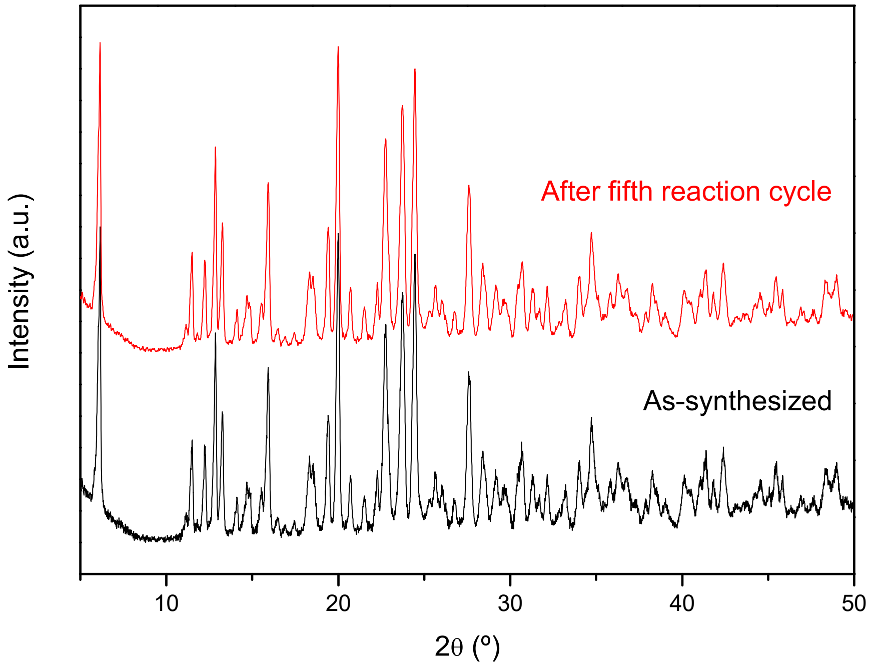

Figure S4. 6. XRD of Zn-URJC-12 material after five reaction cycles.
